# Prognostic significance of heart rate turbulence parameters in patients with chronic heart failure

**DOI:** 10.1186/1471-2261-14-50

**Published:** 2014-04-13

**Authors:** De-Chun Yin, Zhao-Jun Wang, Shuai Guo, Hong-Yu Xie, Lin Sun, Wei Feng, Wei Qiu, Xiu-Fen Qu

**Affiliations:** 1 Department of Cardiology, The First Affiliated Hospital of Harbin Medical University, Harbin 150001, P.R. China; 2 Department of Cardiology, The First Affiliated Hospital of Harbin Medical University, Dazhi Street No.199, Nangang District, Harbin 150001, P.R. China

**Keywords:** Heart rate turbulence, Chronic heart failure, Prognosis

## Abstract

**Background:**

This study is aimed to evaluate the clinical significance of heart rate turbulence (HRT) parameters in predicting the prognosis in patients with chronic heart failure (CHF).

**Methods:**

From June 2011 to December 2012, a total of 104 CHF patients and 30 healthy controls were enrolled in this study. We obtained a 24-hour Holter ECG recording to assess the HRT parameters, included turbulence onset (TO), turbulence slope (TS), standard deviation of N-N intervals (SDNN), and resting heart rate (RHR). The relationships between HRT parameters and the prognosis of CHF patients were determined.

**Results:**

The assessment follow-up period lasted until January 31, 2013. The overall mortality of CHF patients was 9.6% (10/104). Our results revealed that CHF patients had higher levels of TO than those of healthy subjects, but the TS levels of CHF patients were lower than that of the control group. CHF patients with NYHA grade IV had higher HRT_1/2_ rate than those with NYHA grade II/III. There were statistical differences in TS, LVEF, SDNN and RHR between the non-deteriorating group and the non-survivor group. Significant differences in TS among the three groups were also found. Furthermore, CHF patients in the non-survivor group had lower levels of TS than those in the deteriorating group. Correlation analyses indicated that TO negatively correlate with SDNN, while TS positively correlated with SDNN and left ventricular ejection fraction (LVEF). We also observed negative correlations between TS and left ventricular end-diastolic cavity dimension (LVEDD), RHR, homocysteine (Hcy) and C-reactive protein (CRP). Multivariate Cox regression analysis further confirmed that LVEF (≤30%), HRT_2_, SDNN and RHR were independent risk factors which can indicate poor prognosis in CHF patients.

**Conclusions:**

Our findings indicate that HRT may have good clinical predictive value in patients with CHF. Thus, quantifying HRT parameters could be a useful tool for predicting mortality in CHF patients.

## Background

Chronic heart failure (CHF), one of the most common forms of heart failure, is a pathophysiological state in which an abnormality of cardiac function is responsible for the failure of the heart to pump blood at a rate necessary to deliver nutrients to metabolizing tissues [1]. Despite advances in medical and surgical treatments, mortality in CHF patients remains high, and the five-year mortality rate in CHF patients averages 50%, whereas in the end-stage CHF, it may reach 75%; and the sudden death rate accounts for 30 ~ 50% of total mortality [2,3]. Recent studies have confirmed that the prognosis of CHF is uniformly poor, and the accuracy of diagnosis through clinical means alone is often inadequate, particularly in elderly obese people [4,5]. Currently, several studies have indicated that various indices or electrocardiographically derived parameters have been commonly used and tested as prognostic predictors for CHF [6,7].

Clinically, the currently accepted prognostic indicators for CHF mainly consist of heart rate variability (HRV) and LVEF, which may both have some limitations to their own extents [8]. HRV, as one of the oldest Holter-derived risk stratification parameters, is an established parameter for analyzing the performance of autonomic regulation in the sinus node [9]. Nevertheless, it is only mildly inclined to a series of physiological reflexes caused by the external environment and *in vitro* stimulation, and cannot reflect the instantaneous malignant arrhythmias. [10]. As for LVEF, it primarily reflects mechanical LV systolic function, however, in the clinical there were a large number of patients suffering from a high risk of sudden death who only detected a mild to moderate left ventricular function with CHF [11,12]. Therefore, it might be essential to actively explore other useful and valid predictors for CHF sufferers since the existing clinical techniques may be inappropriate to applied as a biomarker of high risk of sudden death in CHF.

Heart rate turbulence (HRT), modulated by the baroreceptor reflex, is a physiological biphasic response of the sinus node to premature ventricular contractions, which involves a short initial acceleration followed by a deceleration of the sinus rhythm due to the baroreflex. [13] There are two numeric descriptors that are usually employed to quantify HRT parameter: one is the turbulence onset (TO), which reflects the initial acceleration of heart rate after a ventricular premature beat (VPB); the other is the turbulence slope (TS), which is used to describe subsequent deceleration of heart rate following a ventricular premature beat [13,14]. Previous studies have shown that HRT assessment might be an important parameter in risk stratification after MI and has been suggested that it could be used as a measure of autonomic dysfunction [15,16]. Clinically, HRT can facilitate the predictive accuracy of nonsustained ventricular tachycardia (NSVT) in identifying patients at risk for serious arrhythmic events and in patients with left ventricular (LV) dysfunction [17]. Recent studies have proved more evidence that HRT parameters may be regarded as surrogate measures for baroreflex sensitivity in clinical and prognostic evaluation in patients with CHF [13,18]. In this study, we evaluated the clinical significance of HRT parameters in predicting prognosis in patients with CHF and determine whether HRT parameters could be considered as markers of the development and progression of CHF.

## Methods

### Ethics statement

The Ethics Committee of the First Affiliated Hospital of Harbin Medical University approved the study design. All patients gave informed consent in written to undergo diagnostic and therapeutic procedure at the time of hospitalization.

### Study design and subjects

The study was performed in patients diagnosed of CHF when admitted to the First Affiliated Hospital of Harbin Medical University from June 2011 to December 2012. Patients enrolled in this study must satisfy all of the following criteria: (1) all patients in the study had to meet the diagnostic criteria for CHF as specified by the 1995 World Health Organization/International Society and Federation Cardiology (WHO/ISFC); [19] (2) there must exist evidence for CHF, such as dyspnea, left/right heart failure, cardiac dilatation and left ventricular ejection fraction (LVEF) < 50%; (3) each patient presented with sinus rhythm also had > 5 ventricular premature beats (VPBs) measured; (4) patients diagnosed with CHF were graded II ~ IV according to the NYHA classification [20]. Patients were excluded based on the following criteria: (1) patients had chronic or persistent atrial fibrillation, an implanted permanent pacemaker or cardiac resynchronization therapy; (2) patients were in the acute phase of congestive heart failure or MI; (4) inability to perform a HRT determination or reliable data analysis; (5) patients had hypertrophic cardiomyopathy, myocarditis, diabetes, heart disease, pulmonary heart disease or history of myocardial infarction within 2 months; (6) patients had liver, kidney and other serious organs diseases; (7) patients took antiarrhythmia drugs other than β-blocker; (8) there existed too much interference in dynamic ECG.

In our present study, 104 CHF patients (72 males and 32 females) were enrolled with a mean age of 61.0 ± 10.0 years (range, 39 ~ 84 years). According to the NYHA classification, these patients were divided into grade II (16 males and 7 females), grade III (20 males and 27 females) and grade IV (27 males and 7 females) groups. There were 37 patients (28 males and 9 females) with ischemic heart disease and 67 patients (35 males and 32 females) with nonischemic heart disease. In addition, 30 healthy controls (21 males and 9 females) with a mean age of 58.0 ± 9.0 years (range, 40 ~ 83 years) were enrolled in the control group after detailed medical examination, which includes medical history taking, physical examination, chest X-ray, echocardiography, Holter monitoring and blood biochemical examination.

### Holter recording and evaluation of HRT parameters

All patients were managed under a uniform and standard assay method. HRT of CHF patients were quantified by TO and TS acquired through the 24-hour 3-lead Holter digital recordings (Syneflash recorder; ELA Medical, Le Plessis Robinson, France) according to a previously published method [13]. HRT was analyzed using the HRT Version 0.6 ~ 0.1 software program (Munich Germany). TO is the difference between the mean of the first two RR intervals after a VPB and the last two RR intervals before the VPB. Positive values for TO indicate deceleration, whereas negative values indicate acceleration of the sinus rhythm. TS, the maximum positive value of the slope of a regression line assessed over any sequence of five subsequent sinus-rhythm RR intervals within the first 20 sinus rhythm intervals after a VPB, are expressed in millisecond per interval RR (ms/RR). TO > or = 0%, TS < or = 2.5 ms/RR were considered abnormal [18]. Based on the previous study, patients in our current study were stratified into three groups: HRT_0_ group with TO < 0 and TS > 2.5 ms/normal-to-normal interval (both factors normal); HRT_1_ group with either TO ≥ 0 or TS = 2.5 ms/RR (1 factor abnormal); HRT_2_ group with TO ≥ 0 and TS ≤ 2.5 ms/RR (both factors abnormal) [18]. For the entire study population, time-domain measurements of heart rate variability (HRV), including mean N-N intervals, standard deviation of N-N intervals (SDNN), and resting heart rate (RHR), left ventricular end-diastolic dimension (LVEDD) were calculated from the 24-hour Holter ECG recording. The RHR was measured after 5 minutes rest while seated in a quiet room [21].

Two-dimensional and Doppler echocardiographic examinations were performed with a HP5500 ultrasound system (Hewlett Packard, Andover, Mass, USA). In the parasternal long-axis view, M-mode echocardiograms were made of left ventricle from the left hemithorax in short-axis.

### Follow-up

The median duration of the entire study follow-up was 10.5 ± 8.3 months (range, 6 ~ 18 months). All events recorded in the study were adjudicated blindly by an ad-hoc committee on the basis of pre-agreed definitions and procedures. Follow-up information was collected for all patients through outpatient service and phone calls. The end point was prospectively defined as the composite of heart failure and cardiac mortality.

The assessment follow-up period lasted until January 31, 2013. Finally, the overall mortality of CHF patients was 9.6% (10/104). There were 32 CHF patients (26 males and 6 females) exhibiting a deterioration of cardiac function and 62 patients showing no significant deterioration.

### Statistical analysis

Data were expressed as mean ± standard deviation (mean ± SD) unless otherwise specified and qualitative data are presented as percentages. Comparative analyses between the groups were performed with the Student’s t-test for continuous variables and Χ^2^ or Fisher’s exact test for dichotomous parameters. Correlation between autonomic markers was assessed with Spearman correlation coefficient. Survival curves were estimated with the Kaplan-Meier method and compared by the log-rank test. Independent predictors of death were identified with multivariate Cox proportional hazards model and expressed as hazard ratio (HR) with 95% confidence interval (CI). A *P* value of less than 0.10 was considered statistically significant and all tests were two-sided. All analyses were performed using the SPSS 18.0 software (SPSS, Inc., Chicago, IL, USA).

## Results

### Comparison of heart rate turbulence

**Figure 1:**
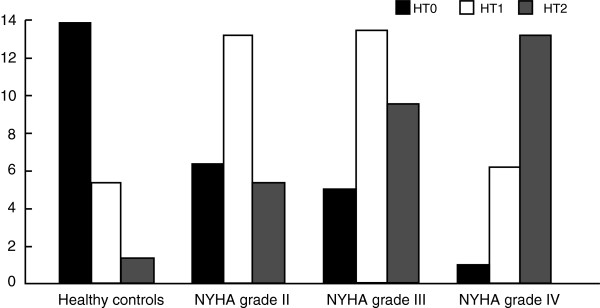
Difference in HRT levels between CHF patients with different NYHA grade and healthy controls.

The differences in HRT between CHF patients and healthy subjects are summarized in Table 1. Our results revealed that CHF patients exhibited a significant reduction in HRT levels. CHF patients had higher levels of TO than those of healthy subjects (0.48 ± 1.58% vs. -1.73 ± 2.58%, *P* < 0.001), but the TS levels of CHF patients were lower than that of the control group (1.88 ± 1.99 vs. 4.41 ± 1.71 ms/RR, *P* < 0.001). There was a significant difference in the rate of HRT_1/2_ between CHF patients and healthy controls (89/104 vs. 22/30, *P* < 0.001). Furthermore, CHF patients with NYHA grade IV had higher HRT_1/2_ rate than those with NYHA grade II/III (*P* = 0.013, 0.051, respectively) (Figure 1).

**Table 1:** Difference in HRT Levels between CHF patients and healthy controls

**Group**	**NYHA classification**	**HRT**_**0**_	**HRT**_**1/2**_	
			**HRT**_**1**_	**HRT**_**2**_
CHF patients	Grade II (n = 23)	6	13	5
	Grade III (n = 47)	8	23	16
	Grade IV (n = 34)	1	10	22
Healthy controls	(n = 30)	22	7	1

*HRT*, heart rate turbulence; *CHF*, chronic heart failure; *NYHA*, the New York Heart Association.

### Relevance factors of HRT

**Figure 2:**
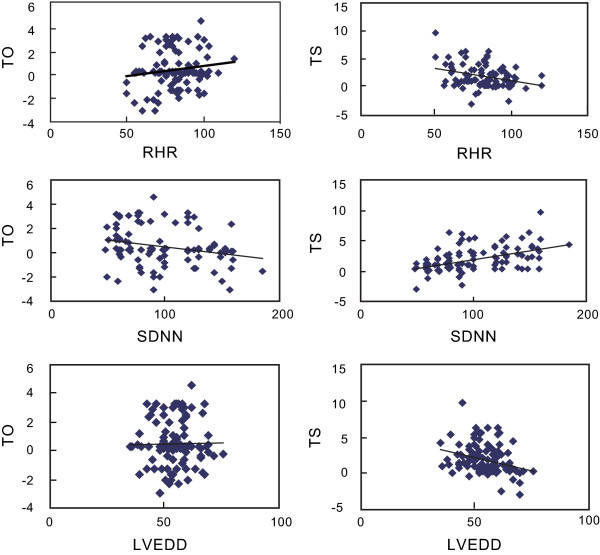
Correlations of HRT parameters (TO and TS) with SDNN, RHR and LVEDD.

Correlation analyses indicated that TO negatively correlated with SDNN (*r* = -0.316, *P* < 0.001), but no associations were found between TO and other factors (all *P* > 0.05). TS positively correlated with SDNN (*r* = 0.270, *P* < 0.001) and LVEF (*r* = 0.365, *P* < 0.001). However, there were negative associations between TS and LVEDD (*r* = -0.313, *P* < 0.001), RHR (*r* = -0.299, *P* = 0.001), Hcy (*r* = -0.198, *P* < 0.001) and CRP (*r* = -0.278, *P* < 0.001). We also observed no correlations with TS to age and TC levels (both *P* > 0.05). Correlations of TO and TS with relevance factors are shown in Table 2 and Figure 2.

**Table 2:** Correlations of TO and TS with relevance factors

**Factors**	**TO**		**TS**	
	***r***	***P***	***r***	***P***
Age	0.760	0.401	-0.600	0.505
LVEDD	0.152	0.090	-0.313	<0.001
LVEF	-0.268	0.060	0.365	0.001
SDNN	-0.316	<0.001	0.270	0.020
RHR	0.211	0.180	-0.299	0.010
TC	0.359	0.301	-0.640	0.305
Hcy	0.314	0.062	-0.198	<0.001
CRP	0.218	0.052	-0.278	<0.001

*TO*, turbulence onset; *TS*, turbulence slope; *LVED*, left ventricular end-diastolic diameter; *LVEF*, left ventricular ejection fraction; *SDNN*, standard deviation of N-N intervals; *RHR*, resting heart rate; *TC*, total cholesterol; *Hcy*, homocysteine; *CRP*, C-reaction protein.

### Comparison of CHF patients with different prognosis

Our results suggested that there were statistical differences in TS, LVEF, SDNN and RHR between the non-deteriorating group and the non-survivor group (all *P* < 0.05, as shown in Table 3). We observed a significant difference in TS among the three groups (all *P* < 0.05). Furthermore, CHF patients in the non-survivor group had lower levels of TS than those in the deteriorating group (*P* < 0.001).

**Table 3:** Comparison of CHF patients with different prognosis

**Factors**	**Non-deteriorating group (n = 62)**	**Deteriorating group (n = 32)**	**Non-survivor group (n = 10)**
Age	64.78 ± 11.92	60.63 ± 10.25	60.12 ± 10.34
TO	0.33 ± 1.64	0.23 ± 1.53	0.95 ± 1.63
TS	2.50 ± 2.61	2.15 ± 1.82^**※**^	0.87 ± 1.33^**※#**^
LVEDD	44.58 ± 10.12	41.24 ± 7.24	26.82 ± 8.86^**※**^
LVEF	56.15 ± 7.17	53.31 ± 9.01	61.01 ± 9.40
SDNN	102.88 ± 36.98	97.28 ± 34.88	95.57 ± 31.66^**※**^
RHR	76.65 ± 15.23	83.48 ± 12.76	89.58 ± 14.07^**※**^
TC	4.56 ± 4.36	4.16 ± 0.16	3.16 ± 0.62
Hcy	8.59 ± 2.06	14.55 ± 4.11	16.95 ± 6.30
CRP	7.71 ± 1.22	11.84 ± 2.96	12.67 ± 5.67

*CHF*, chronic heart failure; *TO*, turbulence onset; *TS*, turbulence onset; *LVEDD*, left ventricular end-diastolic diameter; *LVEF*, left ventricular ejection fraction; *SDNN*, standard deviation of N-N intervals; *RHR*, resting heart rate; *TC*, total cholesterol; *Hcy*, homocysteine; *CRP*, C-reaction protein; ^**※**^*P* < 0.05 compared with the non-deteriorating group; ^**#**^*P* < 0.05 compared with the non-deteriorating group.

### Survival analysis

**Figure 3:**
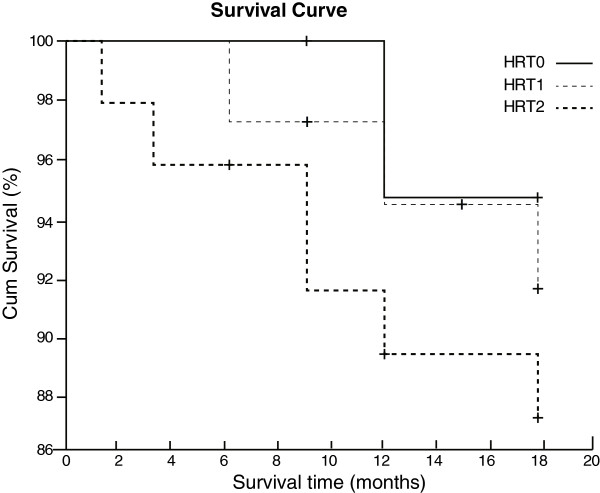
**Survival curve for the survival rate of CHF patients with HRT**_**0**_**, HRT**_**1**_**and HRT**_**2**_**.**

Univariate survival analysis demonstrated that age (≥65), LVEF (≤30%), SDNN (≤100 ms), RHR (≥75 bpm), HRT_1_ and HRT_2_ were associated with poor prognosis in CHF patients (all *P* < 0.05) (Table 4). Multivariate Cox regression analysis indicated that LVEF (≤30%), HRT_2_, SDNN and RHR were independent risk factors for poor prognosis in CHF patients (all *P* < 0.05) (Table 5). Among these risk factors, HRT_2_ was the strongest predictor of CHF with a high hazard risk of 5.12. The survival curve also showed that the survival rate of CHF patients with HRT_0_ and HRT_1_ were higher than those with HRT_2_ (all *P* < 0.05) (Figure 3).

**Table 4:** Univariate survival analysis of HRT parameters with prognosis of CHF patients

	**HR (95% CI)**	***P*** **value**
Age	3.91 (1.03, 19.56)	0.048
HRT_0_	1.34 (0.50, 3.38)	0.648
HRT_1_	3.78 (1.86, 7.01)	0.010
HRT_2_	5.40 (2.65, 25.48)	0.015
LVEF	4.96 (1.83, 17.20)	0.020
LVEDD	2.70 (1.08, 7.36)	0.038
SDNN	3.95 (1.11, 14.02)	0.034
RHR	4.04 (1.39, 20.95)	0.015

*HRT*, heart rate turbulence; *CHF*, chronic heart failure; *HR*, hazard risk; *LVEF*, left ventricular ejection fraction; *LVEDD*, left ventricular end-diastolic diameter; *SDNN*, standard deviation of N-N intervals; *RHR*, resting heart rate.

**Table 5:** Multivariate cox regression analysis of HRT parameters with prognosis of CHF patients

	**HR (95% CI)**	***P*** **value**
HRT_1_	5.12 (1.53, 16.37)	0.022
HRT_2_	4.68 (1.54, 16.78)	0.030
LVEF	1.37 (0.84, 2.13)	0.123
LVEDD	3.69 (1.32, 11.46)	0.046
SDNN	3.18 (1.69, 17.43)	0.034
RHR	1.84 (0.77, 2.46)	0.152

*HRT*, heart rate turbulence; *CHF*, chronic heart failure; *HR*, hazard risk; *LVEF*, left ventricular ejection fraction; *LVEDD*, left ventricular end-diastolic diameter; *SDNN*, standard deviation of N-N intervals; *RHR*, resting heart rate.

## Discussion

HRT is a baroreflex-mediated biphasic reaction of heart rate in response to premature ventricular beats [22]. Generally, abnormal HRT is widely accepted to be indicative of potential autonomic dysfunction or impaired baroreflex sensitivity in patients due to variety of disorders; it may also reflect changes in autonomic nervous system induced by different therapeutic modalities such as drugs, revascularization, or cardiac resynchronization therapy [15,23]. More importantly, impaired HRT has been demonstrated in identifying patients at high risk for all-cause mortality and sudden death, particularly in post-infarction and congestive heart failure patients [24]. Notably, HRT have a higher specificity and sensitivity as compared to some other detection methods since it is a bi-physiological response of the interaction between sinus and ventricular contraction that reflect the sensitive outcome of extremely weak endogenous stimuli triggering reflex regulation [25,26]. In recent decades, increasing number of clinical applications in HRT have gained global popularity as well as considerable successes, and it has been previously described as an independent predictor of mortality in patients with cardiovascular diseases [27,28].

Generally, HRT is described as the acceleration and subsequent deceleration of sinus rate [29]. Abnormal HRT has a well-established role in the stratification of post-infarction and heart failure in patients [30]. However, current emphasis on the medical application of HRT is far from perfect, and its function has not been fully assessed. In this study, we evaluated the relationships between HRT parameters and prognosis in CHF patients to explore the predictive values of HRT parameters in the development and progression of CHF. This study revealed that CHF patients exhibited a significant reduction in HRT levels with higher TO levels and lower TS levels than the healthy subjects, suggesting that the HRT levels may be closely related to the development and progression of CHF. Although the exact reasons for HRT reduction in patients with CHF are not fully understood, a potential explanation could be that CHF patients may suffer from myocardial deformation or damages in the terminal receptors, and abnormal sympathetic and vagal afferent stimulation, which may lead to dullness of baroreflex, cause decreased HRT or disappearance of HRT after VPBs in patients with organic heart disease [31]. Sredniawa et al. demonstrated that CHF patients were associated with more abnormal average values of HRT parameters, as a marker of the autonomic regulation in the heart, might play important roles in the progression toward end-stage heart failure or all-cause mortality of CHF patients [18]. Analysis of HRT is an easy and innocuous way to assess the parasympathetic nervous system, and it can be used as a prognostic marker both in ischemic heart disease and heart failure; in some cases, it even has a better correlation than LVEF [32]. We also found a significant difference in HRT_1/2_ rate between CHF patients and healthy controls. Furthermore, CHF patients with NYHA grade IV had a higher positive rate of HRT_1/2_ than those with NYHA grade II/III, revealing that HRT was correlated with the severity of CHF. The observation of more depressed HRT in patients with advanced NYHA functional class is also consistent with previous reports and studies [33]. In addition, our study also found statistical differences in TS, LVEF, SDNN and RHR between the non-deteriorating group and the non-survivor group; significant differences in TS among the three groups were also observed. Moreover, correlation analyses indicated that both the TS and TO were significantly correlated with SDNN and that only TS was significantly correlated with LVEF. These findings revealed that TS could be considered as a stronger predictor of hospitalization for worsening heart failure and death in CHF patients than LVEF, SDNN and RHR. The mechanisms of the decreased TS in CHF patients are not clear; it is likely that the TS is more susceptible to CHF events because it correlates with both the left ventricular function and the cardiac autonomic status [34]. Consistent with our results, a previous study suggested that the TS appears to be a more suitable prognostic marker for CHF death and hospitalization than LVEF and SDNN, which further confirmed that HRT serves great predictive value in the prognosis of CHF [35]. Univariate survival analysis demonstrated that age (≥65), LVEF (≤30%), SDNN (≤100 ms), RHR (≥75 bpm), HRT_1_ and HRT_2_ were associated with poor prognosis of CHF patients. Multivariate Cox regression analysis indicated that LVEF (≤30%), HRT_2_, SDNN and RHR were independent risk factors for poor prognosis of CHF patients. Among these risk factors, HRT_2_ (the combination of abnormal TO and TS) was the strongest predictor of CHF with a high hazard risk of 5.12 after CHF and remained highly significant after adjustment for LVEF and other clinical risk factors. The combination of abnormal TO and TS may result in tachycardia which would cause decreased heart efficiency in increased myocardial oxygen consumption, decreased myocardial perfusion during myocardial relaxation and diastolic, thereby leading to myocardial injury and aggravating or worsening heart failure [36]. Nevertheless, some limitations of this study should be acknowledged. Firstly, a lack of image analysis software to automatically identify and analyze the matched VPBs, as well as HRT, limited the accuracy in the prognostic significance of HRT in CHF patients. Secondly, HRT level is easily affected by various factors, therefore certain limitations still remain in the sensitivity, specificity and positive predictive value of HRT in CHF patients. Thirdly, due to the short follow-up time and the relatively small sample size, the correlation between HRT and CHF requires further verification.

## Conclusions

Our findings indicated that HRT may have good clinical predictive values in patients with CHF. Thus, quantifying HRT parameters could be useful tool in predicting mortality in CHF patients. However, due to the limitations mentioned above, further detailed studies with larger sample size and longer follow-up periods are still required to confirm our results.

## Competing interests

The authors have declared no competing interests among themselves.

## Authors’ contribution

YDC carried out the experiments and drafted the manuscript. WSJ, GS and FW analyzed the data. XHY, SL and QW contributed the material and the analysis tools. YDC and QXF participated in the design of the study and performed the statistical analysis. QXF conceived the study, and participated in its design and coordination and helped draft the manuscript. All authors read and approved the final manuscript.

## Pre-publication history

The pre-publication history for this paper can be accessed here:

http://www.biomedcentral.com/1471-2261/14/50/prepub
